# Gender differences in wage expectations

**DOI:** 10.1371/journal.pone.0250892

**Published:** 2021-06-02

**Authors:** Ana Fernandes, Martin Huber, Giannina Vaccaro

**Affiliations:** 1 Institute New Work, Business School, Berner Fachhochschule, Bern, Switzerland; 2 Department of Economics, University of Fribourg, Fribourg, Fribourg, Switzerland; 3 Center for Econometrics and Business Analytics, St. Petersburg State University, St. Petersburg, Russia; 4 Swiss Centre for Expertise in the Social Sciences (FORS), Lausanne, Vaud, Switzerland; 5 Department of Economics, University of Lausanne, Lausanne, Vaud, Switzerland; TED University, TURKEY

## Abstract

Using an own survey on wage expectations among students at two Swiss institutions of higher education, we examine the wage expectations of our respondents along two main lines. First, we investigate the rationality of wage expectations by comparing average expected wages from our sample with those of similar graduates; further, we examine how our respondents revise their expectations when provided information about actual wages. Second, using causal mediation analysis, we test whether the consideration of a rich set of personal and professional controls, inclusive of preferences on family formation and number of children in addition to professional preferences, accounts for the difference in wage expectations across genders. Results suggest that both males and females overestimate their wages compared to actual ones and that males respond in an overconfident manner to information about realized wages. Personal mediators alone cannot explain the indirect effect of gender on wage expectations; however, when combined with professional mediators, this results in a quantitatively large reduction in the unexplained effect of gender on wage expectations. Nonetheless, a non-negligible and statistically significant direct (or unexplained) effect of gender on wage expectations remains in several, but not all specifications.

## 1 Introduction

Marked differences remain in today’s labor market concerning the professional paths of men and women. [1], for example, survey the research on the gender wage gap and find that, despite considerable gender wage convergence during the period 1980-2010, that process was much less pronounced at the top of the wage distribution. Further, it was the explained part of the gap that declined, indicating gains in education and experience of women relative to men, whereas the unexplained part of the gap did not change much.

Given the well-established and well-known gender wage gap, as well as the different paths that women and men typically follow in the labor market, rational and forward looking men and women, when asked about wage expectations about their own individual future, would generate forecasts in line with the actual gender wage gap. The goal of our paper is, on the one hand, to examine whether this expectational wage gap is rational, in the sense of matching actual wages from comparable groups in the population, and, further, in the way that our respondents react to information about actual wages; on the other hand, we test whether the gender differences in wage forecasts of our respondents are explained by differences in their professional and personal preferences. Specifically, once we control for how individuals see themselves looking forward, not only professionally but also along personal dimensions, we want to know whether or not gender remains as a residual source of wage expectations.

Averaging the wage expectations from our respondents and contrasting them with averages of actual wages from similar population groups in the labor market, we find that both men and women overestimate wages. To gain further insight into the formation of wage expectations, we perform an experiment to examine how our respondents react to information about actual wages. While women do not change their expectations, males react in an overconfident way to the information provided.

As commonly found in the literature, it is also the case that our male and female respondents report expecting different unconditional wages, with men having higher average wage expectations than women. Our second contribution to the literature is to examine whether this commonly found expectational wage gap vanishes once we control for a broader and more comprehensive set of controls, including detailed answers on professional and personal preferences, the latter inclusive of questions on family formation and desired number of children. Using inverse probability weighting methods in the context of causal mediation analysis to estimate the effects of gender on expected wages, we find that the broader set of covariates attenuates the direct effect of gender on wage expectations. Nonetheless, a non-negligible and statistically significant direct (or unexplained) effect of gender on wage expectations remains in several, but not all specifications.

The paper is organized as follows. Section 2 provides a comprehensive overview of the related literature. Section 3 introduces our data set. Section 4 outlines the methodological framework for decomposing the expectational wage gap. Results are presented in Section 5 and Section 6 concludes.

## 2 Literature review

In order to simplify the exposition and facilitate the connection with our paper, we divide our overview of the literature into two strands: one that attempts to gauge the rationality/accuracy of wage expectations, and the other investigates the reasons why wage expectations systematically vary with gender.

Forward looking, rational economic agents will chose their education and labor market path taking into account the expected rewards associated with different possibilities. Therefore, the accuracy of wage expectations is crucial for efficient decision making at the educational and human capital accumulation stages. Consequently, the literature on wage expectation has focused on the accuracy of those expectations and on how individuals update their expectations when new information is provided.

[2], for example, showed that students’ expectations corresponded to a high degree with the performance of earlier cohorts in the labor market. He further showed that expected income differences between occupations have an influence on the choice of education, assuming a limited set of educational alternatives. [3] find that students whose expected returns from nursing college education were higher were more likely to later enroll in nursing college. In this line of research, great heterogeneity of student wage expectations across fields of study and individual characteristics was well documented. In a reference study, [4] asked high school and college undergraduate students to complete a computerized detailed questionnaire eliciting beliefs about income levels attained under different schooling levels as well as respondents’ beliefs about current earnings distributions. They found that students were capable of making realistic estimates of future incomes and also that most of them expected to have positive college returns. ([5] collected data on expected wages from students of the Economics Department of the Bern University of Applied Sciences using a computer-assisted methodology similar to the one used in [4]).

Similarly, [6] found that student expectations were reasonably aligned with income realizations despite considerable individual variability linked to personal traits (such as year and field of study). More recently, in a series of contributions, [7, 8] documented that college students update their expectations in a logical way when provided information about future earnings despite despite initially having biased estimates of the population of earnings. (Similarly, [9] find that students’ initial wage expectations are highly biased, underestimating actual salaries by 18 percent, but that this bias is largely due to misconceptions of the progressive German income tax).

Regarding gender differences in wage expectations, the overall view in the literature is that men and women have different own wage expectations despite having good information about earnings of their peers ([10]). Despite both men and women overestimating their earnings, women’s expectations seem to be more realistic when entering the labor market ([6, 11]). Furthermore, this gender gap closely resembles actual wage differences, prevails across subgroups and along the entire wage distribution ([12]). ([13] find that women appear to have less accurate wage expectations concerning the long term).

This strand of the literature has also examined whether gender differences in wage expectations remain even after current salary information is provided. After interviewing almost 100 students from Business and Economics Departments at a mid-size American university, [14] reports the persistence of the common gender expectational wage gap even after all interviewed students were given information about combined salaries of males and females. Likewise, [15] examined expectations revisions over time using a Dutch panel where information on income expectations for the same household is available in consecutive years. Comparing expected and realized income changes for the same time period, they found that, on average, future income growth was significantly underestimated. In particular, people whose income decreased in the recent past tended to be too pessimistic. Negative transitory incomes were too often considered to be permanent.

We advance this literature by comparing students’ wage expectations with realized wages for similar groups of graduates while simultaneously quantifying the prevailing expectational gender wage gap. Further, through an information experiment, we directly analyze how information on average income alters wage expectations along gender lines. While the results from the information experiment cannot be directly connected to the (size of the existing) expectational wage gap found in our sample, they nonetheless help shed light on the underlying processes of expectation formation, in line with the literature.

Side by side with the literature testing the rationality and accuracy of wage expectations, a different strand of work has attempted to provide reasons why expectations vary across gender to begin with. The literature has overwhelmingly confirmed that males expect higher wages than females (see e.g. the pioneering work of [14, 16, 17]). This gender gap is present even before individuals start their professional career and increases over the life-cycle ([18]). Moreoever, it is not limited to the American context ([12, 19, 20]).

Despite these gender differences in wage expectations having been documented extensively in the literature, the key question is indeed how gender differences in wage expectations are explained. Multiple studies provide different answers. We discuss next five of the main causes that might drive gender differences in wage expectations, and determine how family and career expectations contribute to explain this gap.

First, individual characteristics as well as job preferences may lead men and women to chose different jobs and make different career decisions. For example, in [18], men and women are conscious of the pay implications that choosing a female- or a male-dominated job will imply. Along similar lines, [21] found that men have higher promotion expectations for male- and neutral-oriented jobs than their female counterparts. (Existing studies have not been limited to female-male job classifications, however. [22] confirm that women have consistent lower wage expectations than men across different education programs such as STEM and non-STEM fields. Nonetheless, they found that differences in wage expectations were not explained by the probability of choosing a STEM major).

Second, psychological features such as self-perceptions, self-esteem and self-efficacy have also been examined. The psychological literature, in particular, is very rich regarding self-related theories and has shown that wage expectations and self-views are correlated with job attributes and pay expectations. In [23], for example, female students have lower wage expectations and are less confident than males. These authors found that about 7.7 percent of the gender gap in wage expectations is attributable to higher overconfidence of males. (Contrasting with their findings, [24] found that gender differences in self-views favor females in China. They attribute this evidence to their sample—in which most women hold a career track position and have longer average years of education than the national average in China).

A third explanation for gender differences in wage expectations is rooted in gender differences in attitudes towards preference for competition and negotiation skills [25]. Using a sample of about 1500 Swiss lower-secondary school students in Switzerland, [26] found that, at all levels of the ability distribution, willingness to compete is associated with choosing more challenging options which, in turn, leads to higher-paid careers. This behavior towards competition may carry over to other fields and situations, for example to salary negotiations and negotiation skills [27]. Nonetheless, [28, 29] find that gender differences in risk-aversion, over-confidence and competitiveness do not suffice to close the gender expectational wage gap among college students in a North-American University.

A fourth explanation examines factors such as career referents, stereotypes, social comparisons, and perceived discrimination or perceptions about pay standards. [30] found that women have lower expectations than men even when they identified high-level referents and even when those referents are women. Related to this, [17] show evidence that same-sex comparisons are a stronger predictor of career-entry pay expectations than opposite-sex comparisons. A recent laboratory experiment of [31] showed that stereotypes affect self-beliefs and own performance. It is therefore important to know whether individuals have accurate wage perceptions.

A fifth explanation concerns gender differences in attitudes related to work as well as family values and aspirations. Individuals might be already aware at an early age of their career and family plans, and therefore internalize those in their future decisions in the labor market. As a result, perhaps expecting to work fewer hours or taking jobs that help reconciling family and work ([32]), women may have lower salary expectations, and lower wages compared to men ([33]). [34] documented that child rearing and career break expectations accounted for about 10% of the gender wage gap in the UK. For these authors, because women have child rearing preferences and expect to take a career break, they reduce their search and expect a lower wage. As consequence, they are less willing to move across jobs, and more likely to obtain a lower wage. Related results were found by [35] when studying the impact of workplace preferences on the expected and actual gender wage gaps. They show that gender differences in job preferences (i.e. work flexibility, job stability, hours worked potentially compatible with child rearing), as well as preferences for high earnings growth explain a sizable part (about 35 log points) of the gender gap in expected earnings early in the life cycle (expected wage at the age of 30 years). ([11] also pointed out that wage expectations are affected by time preferences for childrearing but also for conciliating weekly hours worked with family responsibilities. Using very detailed information on career plans and earning expectations of college business school seniors, [36] provide evidence women expect to work fewer years than men. However, gender differences in expected earnings have no effect on the number of years that women expect to work in the labor market).

In our paper, we paid particular attention to the last explanation in the list above. We began by constructing an own survey of undergraduate business, business and IT, and economics students from two Swiss institutions of higher education. Our survey includes detailed questions on individuals’ perceived professional and personal path moving forward. It asks respondents about their preferences over job attributes, industries and occupations, as well as over family formation, number of children and intended degree of labor market attachment in the presence of children. We directly contribute to the literature comparing the role of these covariates in gender differences in wage expectations. Further, we approach this problem with more sophisticated empirical methods allowing us to appropriately handle endogeneity and nonlinearities and perform a causal mediation analysis to identify the role of gender in determining wage expectations.

## 3 Data

Our data was collected by running a detailed survey among undergraduate students in two Swiss institutions of higher education, namely the Business School of the Bern University of Applied Sciences (BUAS, BFH for the German acronym), and the Faculty of Economic and Social Sciences of the University of Fribourg.

Universities of Applied Sciences are institutions administering tertiary education of a more applied nature. They typically provide the last educational segment to students who followed the apprenticeship track in their upper secondary education (the latter comprising school grades 10 through 12) and who still wish to further their education. Students who followed the university track in upper secondary education go into conventional universities, with a less applied focus.

The university (or, equivalently, the Gymnasium) track is normally reserved for students with higher academic achievements. In Switzerland, roughly two thirds of students follow the apprenticeship track whereas the remainder one third continue school in the university track. The apprenticeship system is a hallmark of the German speaking economies (Switzerland, Germany, Austria) and a large share of the population follows this more applied educational path. As early as their seventh grade in school, at the beginning of lower secondary education, students are put into different educational tracks as a function of their past performance to date.

The answers to our survey were collected on paper. Individual classes were visited and the survey was presented to students for immediate completion and collection. Data collection took place mostly during the first week of the Spring semester of 2017 in order to maximize the response rate. All undergraduate classes of the Business Administration (BBA) and Business and IT (BWI) degrees were visited at BUAS; at the University of Fribourg, respondents attended one of two large introductory statistics classes in the Economics program. Respondents in Fribourg were enrolled in three different degree programs: Bachelor in Economics (VWL), Business Administration (BBA) and Communications (KOMM).

The questionnaires comprised three groups of questions. Students were first asked about general information, such as age, gender, nationality, degree chosen and whether they were enrolled in a part- or full-time program. This was followed by two separate blocks of questions about professional and personal matters.

The professional section asked students about their preferences regarding a variety of job attributes and characteristics of the work environment, whether they intended to work full or part-time upon completion of their degree, industry and occupation where they envisioned working, the expected wage upon completion of the degree and three years upon graduation, if they would rather hold a management or a consulting/supporting position.

The personal section inquired about the intent of forming a family, number of desired children and intended degree of labor market participation in the presence of children, the labor market attachment of the respondent’s parents during different stages of childhood (daycare, Kindergarten and primary school ages of the respondent), type of residence, family composition, and educational attainment of both parents, among other questions. In total, we gathered 865 questionnaire responses from both educational institutions combined.

While all questionnaires had these three groups of questions, the order in which professional and personal questions appeared was randomized. In the control version of the questionnaire, general questions were followed by professional questions, with the personal block appearing at the end. In another version of the questionnaire, the second group of questions was about personal matters and the professional block only showed at the end. We labeled this version as “the different order” version. A third version of the questionnaire retained the question order of the control version but introduced a bar graph with information on monthly gross income in the private sector, according to age and gender. This is the “information” version of the survey. It is important to note that this information was not necessarily helpful for forming expectations about one’s own wage directly upon finishing university of three years thereafter. This because it neither focused on university graduates nor on years in the labor market. The information version of questionnaire can be found in Appendix A in S1 Appendix. (As indicated above, the control version was obtained by eliminating the bar graph from page 1. The “different order” questionnaire swapped question groups A2 and A3, which were relabeled accordingly).

**Fig 1:**
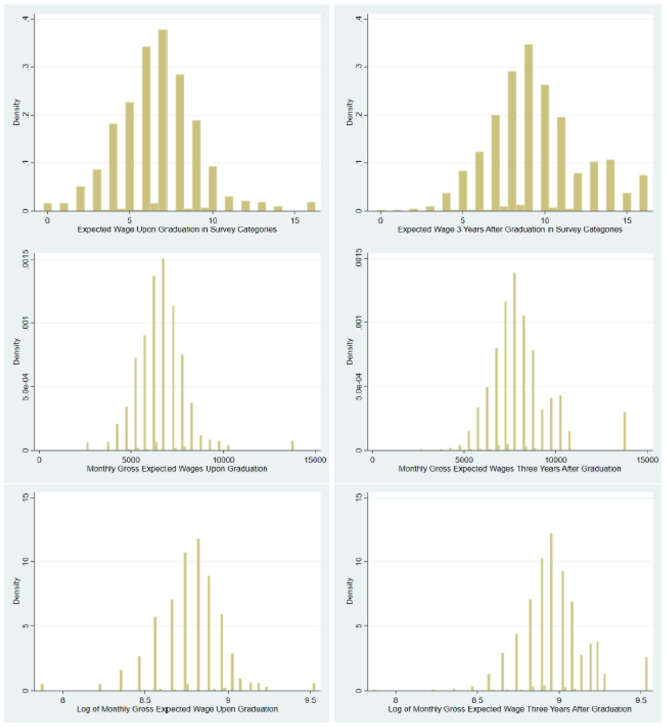
Histograms of expected wages in own sample.

Fig 1 presents histograms of the expected gross monthly wage variables in our sample, as follows. The two panels in the top row present expected wages in survey categories. Those went from 0 (less than CHF 3’500) to 16 (more than CHF 11’000). When respondents selected two adjacent categories, we recorded this answer as the average of those categories. The figures on the left column refer to expected wages upon graduation and those on the right column refer to those three years afterwards. Typical of income data, expected wages display right-skewness.

Categorical data were converted into monthly values using a procedure in [19]. (The average of the lower and upper limits of the wage survey categories were assigned to respondents that had selected those categories; for the lower interval (average gross wage less than CHF 3’500), the assigned wage was 0.75 of the interval’s upper limit and, finally, for the top interval (average gross wage more than CHF 11’000), the assigned amount was 1.25 times the lower limit). The resulting expected wages “in levels” are displayed in the middle row of Fig 1. Finally, the bottom row of Fig 1 presents the logs of the wages in levels, therefore the logs of the wages displayed in the middle row of the figure.

In the comparison of expected wages from our students to those of comparable graduates, we resorted to the FH-Lohnstudie (https://www.fhschweiz.ch/fh-lohnstudie), a yearly survey of wages of alumni of all Swiss Universities of Applied Sciences, thus inclusive of the BUAS. Survey participation is voluntary and elicited by email, and further completed in an electronic format. We focused on the 2017 FH-Lohnstudie edition since this was the same year of our own data collection. The FH-Lohnstudie provides only average wages and no detailed individual data. For this reason, we are not able to plot the distribution of realized wages for that survey.

## 4 Methodological framework

The decomposition of wage gaps across gender aims at disentangling the total gap into an explained component that can be attributed to differences in observed labor market relevant characteristics and an unexplained remainder. In addition to the classical linear decomposition of [37, 38], non-parametric decomposition methods have for instance been proposed by [39, 43], as well as methods for decompositions at quantiles rather than means, see [39, 44–49]. Despite these analytical advances, such progress in estimation methods stands in contrast to the widespread ignorance of identification issues; in particular, the endogeneity of observed characteristics, as for instance pointed out in [50].

Following [51, 52], we formulate the decomposition in the context of a causal model for mediation analysis which allows explicating endogeneity issues. Mediation analysis, as for instance discussed in [53], aims at disentangling the causal mechanisms through which an explanatory variable affects an outcome, with mediators being intermediate outcomes lying on the causal pathway between the explanatory variable and the outcome. Applied to gender decompositions, on the one hand, gender is the explanatory variable at the beginning of any individual’s causal chain affecting expectational wage, because it is determined at or prior to birth. Choice of study program, career preferences, and family plans, on the other hand, are mediators (often referred to as observed characteristics in the wage decomposition literature), because they occur later in life and are thus potentially influenced by gender, while the mediators themselves likely affect wage expectations. Given this causal structure, the explained component in the decomposition literature corresponds to the indirect effect of gender on wage expectations that operates through these mediators. Conversely, the unexplained component equals the direct effect of gender on wage expectations that operates through unobserved mediators like unmeasured personality traits.

**Fig 2:**
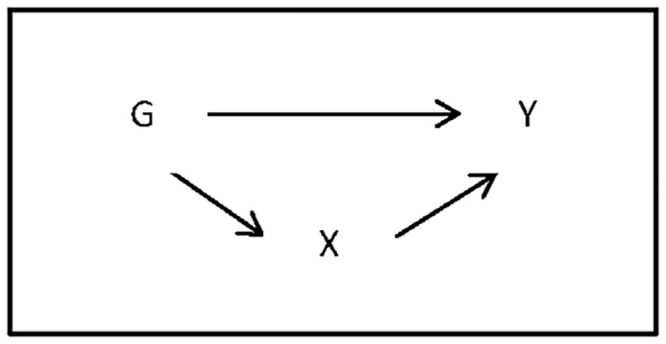
Graphical representation of the decomposition.

More formally, let *G* denote a binary dummy for gender, *Y* the wage expectations outcome and *X* a vector of observed mediators. *G* may affect *Y* indirectly via its effect on *X*, i.e. by a causal mechanism related to observed characteristics. For instance, gender might have an effect on expected wages because females and males target different job types. *G* might influence *Y* also directly, i.e. through factors not observed in *X*. A graphical representation of this causal framework is given in Fig 2, with arrows representing causal effects: *G* influences *Y* either through *X* or directly. For defining the effects of interest, we denote by *Y*(*g*) and *X*(*g*) the potential outcomes and mediators when exogenously setting gender *G* to value *g*, with *g* ∈ {1, 0}. (See for instance [54] for an introduction to the potential outcome framework). Then, *E*(*X*(1)) − *E*(*X*(0)) gives the average causal effect of *G* on mediators *X*, while *E*(*Y*(1)) − *E*(*Y*(0)), corresponds to the (total) average causal effect of *G* on *Y*, represented by the sum of direct and indirect (i.e. operating through *X*) effects.

As in [55, 56] (among many others), we further refine the potential outcome notation to adapt it to the mediation framework: Let *Y*(*g*) = *Y*(*g*, *X*(*g*)), which explicates that the potential outcome is influenced by the group variable both directly and indirectly via *X*(*g*). We can thus express the total effect of *G* on *Y* as *E*(*Y*(1)) − *E*(*Y*(0)) = *E*[*Y*(1, *X*(1))] − *E*[*Y*(0, *X*(0))] in order to decompose the latter into direct and indirect effects. That is, the difference in potential outcomes due to altering *X*(1) to *X*(0) while keeping gender fixed at *G* = 1 yields the indirect effect (denoted by *ψ*), while modifying gender *G* and keeping the characteristics constant at *X*(0) yields the direct effect (*η*). Summing both up gives the total causal effect, as formally expressed below:

$$\begin{array}{c} E\left[Y\left(1,X\left(1\right)\right)\right]-E\left[Y\left(0,X\left(0\right)\right)\right]=\underset{\psi }{\underset{︸}{E[Y(1,X(1))]-E[Y(1,X(0))]}}\\ +\underset{\eta }{\underset{︸}{E[Y(1,X(0))]-E[Y(0,X(0))]}} \end{array}$$ (1)

Depending on whether (the wage expectations of) males or females are considered as reference group with *G* = 1, the magnitudes of *ψ* and *η* may differ due to interaction effects between *G* and *X*, i.e. effect heterogeneities in gender. In the application provided in Section 4, we present the results when considering both females and males as reference group.

The Oaxaca-Blinder decomposition consistently estimates the indirect and direct effects as the explained and unexplained components, respectively, when both *G* and *X* are exogenous. This rules out confounding of the gender-outcome, gender-mediators, or mediators-outcome relationship. In addition, the decomposition requires the wage expectations to be linear in the mediators is also required. Assumption 1 formalizes these restrictions.

**Assumption 1 (sequential independence)**:

(a) {*Y*(*g*′, *x*), *X*(*g*)} ⊥ *G* for all *g*′, *g* ∈ {0, 1} and *x* in the support of *X*,
(b) *Y*(*g*′, *x*) ⊥ *X*|*G* = *g* for all *g*′, *g* ∈ {0, 1} and *x* in the support of *X*,
(c) *Y*(*g*, *X*) is linear *X* for *g* ∈ {0, 1},

where ‘⊥’ denotes statistical independence. Under Assumption 1(a), *G* quasi-random, i.e. there are no variables affecting both *G* on the one hand and *Y* and/or *X* on the other hand. Under Assumption 1(b), observed characteristics like education quasi-random within gender, i.e. given *G*, so that there are no affecting both *X* and *Y*. (Assumptions 1(a) and 1(b) could be relaxed to mean independence when considering average wage gaps, while full independence is required for decompositions of quantiles). Assumption 1(c) imposes potential outcomes to be linear in *X*.

As also discussed in [52], under Assumption 1(a), *E*(*X*(*g*)) = *E*(*X*|*G* = *g*), while under Assumptions 1(a), 1(b), and 1(c), *E*[*Y*(*g*, *x*)] = *E*(*Y*|*G* = *g*, *X* = *x*) = *c*_*g*_ + *xβ*_*g*_, with *c*_*g*_ denoting a gender-specific constant and *β*_*g*_ a vector of gender-specific coefficients on *X*. By iterated expectations, *E*[*Y*(*g*, *X*(*g*′))] = *c*_*g*_ + *E*(*X*|*G* = *g*′)*β*_*g*_ for *g*, *g*′ ∈ {0, 1}. Therefore,

$$\begin{array}{ccc} \psi & = & E\left[Y\left(1,X\left(1\right)\right)\right]-E\left[Y\left(1,X\left(0\right)\right)\right]=\left[E\left(X|G=1\right)-E\left(X|G=0\right)\right]\beta_{1}, \end{array}$$ (2)

$$\begin{array}{ccc} \eta & = & E\left[Y\left(1,X\left(0\right)\right)\right]-E\left[Y\left(0,X\left(0\right)\right)\right]=c_{1}-c_{0}+E\left(X|G=0\right)\left(\beta_{1}-\beta_{0}\right). \end{array}$$ (3)

The probability limits of the explained and unexplained components in the linear Oaxaca-Blinder decomposition correspond to the left hand expressions in (2) and (3), respectively.

In this paper, we also consider a semiparametric propensity score weighting approach for causal mediation analysis suggested in [57], that improves on the Oaxaca-Blinder decomposition in two dimensions. First, it does not impose linearity of the outcome in the mediators and second, it allows controlling for observed confounders not influenced by the treatment, henceforth denoted by *W*. We therefore control for a range of socio-economic variables (including age, nationality, number of siblings, parental education and occupation, material wellbeing) to at least mitigate the endogeneity of gender (which is not necessarily random in the two institutions considered) and the mediators. Among *W* we also include the randomized version of the questionnaire, i.e. indicators for whether the order of professional and personal questions was reversed or whether a graph with information on median monthly gross earnings by age and gender were shown. While the questionnaire version is not related to *G* due to randomization, it might potentially affects both *X* and *Y*.

Formally, our estimation approach is consistent under Assumption 2, which has among others also been considered in [58].

**Assumption 2 (sequential conditional independence)**:

(a) {*Y*(*g*′, *x*), *X*(*g*)} ⊥ *G*|*W* for all *g*′, *g* ∈ {0, 1} and *x* in the support of *X*,
(b) *Y*(*g*′, *x*) ⊥ *X*|*G* = *g*, *W* = *w* for all *g*′, *g* ∈ {0, 1} and *x*, *w* in the support of *X*, *W*,
(c) Pr(*G* = 1|*X* = *x*, *W* = *w*) > 0 and 0 < Pr(*G* = 1|*W* = *w*) < 1 for all *x*, *w* in the support of *X*, *W*.

Assumptions 2(a) and (b) require that conditional on *W*, no unobserved confounders either jointly affect *G* and *Y*, *G* and *X*, or *X* and *Y* given *G*. We acknowledge that this may not hold in our empirical application presented further below, given the limited number of observed control variables. Yet, including control variables likely improves upon conventional wage decompositions that do not account for any form of confounding. Assumption 2(c) is a common support condition, requiring that the conditional probability of belonging to the reference group (*G* = 1) given *X*, *W* is larger than zero, while the conditional probability given *W* must neither be zero nor one. The latter restriction means that for each value of *W*, there exist both females and males in the population.

Under Assumption 2, it follows from the results on identification of direct and indirect effects by inverse probability weighting (IPW) in [57] that

$$\begin{array}{ccc} \psi & = & E[\frac{Y\cdot G}{\mathrm{Pr}(G=1|W)}]-E[\frac{Y\cdot G}{\mathrm{Pr}(G=1|X,W)}\cdot \frac{1-\mathrm{Pr}(G=1|X,W)}{1-\mathrm{Pr}(G=1|W)}], \end{array}$$ (4)

$$\begin{array}{ccc} \eta & = & E[\frac{Y\cdot G}{\mathrm{Pr}(G=1|X,W)}\cdot \frac{1-\mathrm{Pr}(G=1|X,W)}{1-\mathrm{Pr}(G=1|W)}]-E[\frac{Y\cdot (1-G)}{1-\mathrm{Pr}(G=1|W)}]. \end{array}$$ (5)

In our application, we estimate Pr(*G* = 1|*X* = *x*, *W* = *w*) and Pr(*G* = 1|*W* = *w*) by probit regressions and *ψ* and *η* by normalized sample analogues of 4 and 5, respectively, implying that the weights of observations within treatment states sum up to one in our sample. Furthermore, we drop observations with estimated propensity scores below 2% (or 0.02) and above 98% (or 0.98) to prevent that some observations receive too extreme weights in the estimation of direct and indirect effects.

In addition to the expectational wage gap decomposition by gender, the empirical analysis investigates whether the randomized questionnaire version affects the wage expectations of females and males differently. The questionnaire version is therefore once regarded as control variable for mediator-outcome confounding in the decomposition, and once as treatment variable to assess its causal effect on wage expectations.

## 5 Results

We begin our empirical analysis by comparing average wages of students in our sample to averages of realized wages from comparable graduates. We do so while focusing on the subsample of graduates from the Bern University of Applied Sciences (BUAS) since a recurrent survey of current wages of alumni of these universities is available. As mentioned earlier, education at a university of applied sciences is generally of a more applied nature. The conventional university or Gymnasium track is reserved for children who obtained higher grades along primary school (first to sixth) grades. For this reason, and conditioning on studying for the same academic degree, graduates from the University of Fribourg are likely to expect higher wages than graduates from the Bern University of Applied Sciences. This is indeed the case, for example, for students in Business Administration: while expected wages upon graduation are virtually identical (roughly 6.9 in terms of salary classes, or CHF 6’950 monthly), they are CHF 7’135 for Bern and CHF 7’493 for Fribourg three years on). For this reason, we focus on BUAS students in the comparison between our own survey and reported wages from the FH-Lohnstudie.

The first two rows of Table 1 contain average gross monthly wages from the FH-Lohnstudie, a yearly survey of wages of alumni of all Swiss Universities of Applied Sciences, thus inclusive of the BUAS. (Respondents are asked to report their current yearly gross wages. In Switzerland, wages are paid in identical monthly amounts except for July and December, when workers receive one and a half times the wage of the other months. Thus, in order to go from yearly wages to monthly payments, we scaled the yearly amounts by thirteen). The reported data concern averages of monthly wages from alumni answers collected in the 2017 survey, the same calendar year as our in-class survey collection.

**Table 1:** Descriptives: Realized wages and wage expectations.

Means	Sample source	Years of Experience	Obs	Male	Obs	Females	% Change Males-Females
Realized	FH-Lohn (2017)	all ages, BBA, BUAS	26	6116.85	18	5667.54	7.93%
Wages	FH-Lohn (2017)	age < 30, BBA, all UAS	111	6099.54	100	5776.46	5.59%
Wage	Fribourg University & BFH	0	497	6858.15	360	6252.43	9.69%
Expectations		3	498	8418.42	360	7546.18	11.56%
(own survey)	Only BFH	0	391	6890.35	249	6424.20	7.26%
		3	392	8430.80	249	7564.76	11.45%
	Treatment decomposition (BFH only)						
	Exposed to information (information treatment)	0	130	6947.12	75	6291.67	10.42%
		3	129	8551.36	75	7553.33	13.21%
	No treatment (control group)	0	137	6889.60	79	6465.19	6.56%
		3	140	8333.04	80	7518.75	10.83%
	Different question order (order treatment)	0	124	6831.65	95	6494.74	5.19%
		3	123	8415.65	94	7613.03	10.54%

Note: Monthly wages, data from the survey of the Association of Universities of Applied Sciences (FH-Lohnstudie), and our own survey.

We focused on two subsamples of FH-Lohn respondents. In the first row (all ages, BBA, BUAS), we have the subset of respondents that is closest to our own (BUAS) subsample: these are respondents who took the same exact Bachelor in Business Administration administered at the BUAS, and who graduated in 2016. Because the sample size is small (26 males and 18 females, corresponding to 41 and 24% of the total population of graduates by gender, respectively), we considered another partition of respondents, namely those who are younger than 30 years old and who likewise graduated with a Bachelor in Business Administration in 2016, but now across *all* universities of applied sciences, not just the BUAS. Average wages for students in this latter group are presented in the second row of the table (age < 30, BBA, all UAS). (There were 161 graduating students at the BUAS in Business Administration in 2016. Of these, 81 were female and 80 male; 90% of the female graduates (73 students) were younger than 30 while the corresponding share for male graduates was 87% (64 students). Thus, the restriction of being younger than 30 years of age imposed in the partition of the FH-Lohn respondents considered in the second row of Table 1 does not greatly affect its comparability with the respondents from the first row).

For males, the reported wages are very similar across the two FH-Lohn groups (the difference between first and second row average wages is just above 17 Swiss Francs). For women, the difference is larger (roughly 109 Swiss Francs), with higher average wages coming from the larger sample (second row). Given the larger sample size, in the comparisons below, we resort to FH-Lohn respondents who graduated in Business Administration across all Universities of Applied Sciences in Switzerland as our reference group for averages of realized wages (second row of Table 1). Rows 3 through 6 include average monthly wages from our own sample (in levels), first from the full sample of BFH and the University of Fribourg combined, and then for BFH alone. The bottom 6 rows partition the BFH into three subsamples: the control subsample, the information treated group (which received information on outside wages in their questionnaire), and the subsample where the order of personal and professional questions was reversed (but which received no outside wage information).

Our data confirm one stylized fact from the literature ([12, 19]), namely the existence of an expectational gender wage gap (rows 3 through 10, last column). In particular, for our overall sample, the expectational gender wage gap is 9.7%, concerning expected wages upon graduation, and 11.6%, for expected wages three years thereafter. Actual wages from FH-Lohn are also in line with the well-established (raw) gender wage gap [35], as can be seen in rows 1 and 2, last column). Using the FH-Lohn, we document that expected wages increase over time, with expectations 3 years ahead of graduation systematically exceeding those for graduation wages (rows 3 through 10, along “Male” and “Female” columns).

For the purpose of comparing realized wages and wage expectations, the two most similar groups in Table 1 concern FH-Lohn survey respondents and the subsample of BFH students in our data. Table 2 computes the percentage excess of the average wage expectations from different BFH subsamples relative to the 2017 average wage from FH-Lohn, the latter from row 2 in the preceding Table. (Since realized wages corresponds to the calendar year immediately after graduation, we perform those comparisons for expected wages upon graduation only—and thus ignore expected wages three years thereafter in this exercise).

**Table 2:** Deviations from Reality: Percentage differences between expected and realized wages.

Gender Wage differences		
	Men	Women
	0 years of experience	
BFH (all groups)	12.97	11.21
Exposed to information (information treatment)	13.90	8.92
No treatment (control group)	12.95	11.92
Different question order (order treatment)	12.00	12.43

Note: Gender wage differences are measured as (*w*^*e*^ − *w*)/*w*, in percentages. Computation based on expected and realized monthly wages from Table 1.

Since the sample size of the FH-Lohn survey is small and survey response is voluntary, it is of course possible that FH-Lohn respondents are self-selected and are therefore not a representative sample of BFH graduates in Business Administration, which we believe our own sample to be. Self-selection could bias average wages in different ways. Individual differences in the opportunity cost of time would suggest that average wages in the FH-Lohn sample are biased down as students with higher wages would be less eager to respond to the survey as their opportunity cost of doing so is higher. However, having a higher wage is also a reason to want to respond so as to show one’s success in life when memories from student life are still very fresh. The net effect of self-selection is therefore not clear. Given this, we interpret the comparison of realized and expected wages below with some caution.

Keeping in mind the possible self-selection in the answers to FH-Lohn, the numbers in Table 2 suggest that both men and women overestimate their wages relative to those of comparable graduates (all entries are positive). The average of BFH wage expectations for males in the full sample exceeds by 12.97% actual earnings of similar graduates in 2017. For females, this gap amounts to 11.21%. The quantitative percent deviation between expected and actual wages is generally larger for men than for women, with the exception of the “order treatment” (last row of Table 2).

Table 2 additionally shows how average wage expectations changed for the group that received outside wage information, as well as for those who received questionnaires with personal questions ahead of professional ones. For information treated males, average expected wages appear to increase while, for females, they decline. The opposite occurs for the “order treatment.” We next proceed to a rigorous analysis of the causal effects of information and questionnaire randomization treatments on wage expectations.

In what follows, we have divided the variables of our dataset into the following categories: control variables *W*, mediators *X*, outcomes *Y* (the expected gross wage category directly after the studies or 3 years later), and *G* for gender.

To investigate whether randomization was successful. Table 3 reports the means of the control variables *W* separately for the control group, for questionnaires with the information treatment, and for those with a reversed order of personal and professional questions. Mean differences between the means of the respective treatment group and control group as well as p-values of mean difference tests are also provided. Balance appears to be decent (albeit not perfect), as only 3 differences are significant at the 10% level and only 1 is at the 1% level.

**Table 3:** Descriptives for experiment.

	control	Treatment: Information			Treatment: Order			missing
	mean	mean	dif	pval	mean	dif	pval	
female	0.39	0.41	0.02	0.59	0.47	0.08	0.06	1
age	23.06	23.25	0.19	0.35	23.25	0.20	0.34	0
Swiss	0.89	0.86	-0.03	0.23	0.89	0.00	0.85	0
has siblings	0.89	0.92	0.03	0.20	0.95	0.06	0.01	5
mom has higher education	0.24	0.20	-0.04	0.22	0.20	-0.04	0.26	8
dad has higher education	0.39	0.40	0.01	0.80	0.37	-0.02	0.63	13
mum worked full time when I was 4-6	0.15	0.16	0.01	0.75	0.14	-0.01	0.68	7
mum worked part time when I was 4-6	0.44	0.45	0.01	0.80	0.41	-0.03	0.42	7
wellbeing	2.41	2.30	-0.11	0.08	2.35	-0.05	0.35	5
home owner	0.45	0.40	-0.06	0.18	0.45	-0.01	0.89	8
program: business admin	0.72	0.71	-0.01	0.82	0.72	-0.00	0.90	2
program: economics	0.04	0.02	-0.02	0.28	0.02	-0.01	0.36	2
program: communication	0.06	0.08	0.02	0.29	0.07	0.01	0.70	2
program: business IT	0.15	0.13	-0.02	0.41	0.14	-0.01	0.70	2
number of observations	298	277			293			

Note: ‘mean’, ‘dif’, and ‘pval’ reports the respective means, mean differences and p-values of the mean differences. ‘missing’ provides the number of missing observations in the respective variable.

Table 4 reports the results of the treatments on either outcome separately for females and males. The first row (‘mean differences’) presents the experimental estimate based on mean differences in outcomes between the respective treatment group and the control observations. (Wage expectations after studying and three years later are not reported for 10 and 9 observations, respectively, that are dropped from the analysis). The second row (‘OLS with controls’) provides the estimated when linearly conditioning on *W* based on OLS to control for any imbalances in the potential confounders. (38 observations with either missings in *W* or *Y* are dropped from the analysis). The third row (‘double lasso’) presents the results when using (double) lasso-based estimation of the treatment propensity score Pr(*G* = 1|*W*) and of the outcome *E*(*Y*|*G*, *W*) to estimate the treatment effect by semiparametric doubly robust estimation (see [59]). To this end, we use the ‘rlassoATE’ command with its default options of the ‘hdm’ package by [60] for the statistical software ‘R’. This method controls for elements in *W* in a data-driven way under the assumption of approximate sparsity, i.e. that relatively few variables suffice for tackling most of treatment-outcome confounding.

**Table 4:** Intervention effects.

	Treatment: Information						Treatment: Order					
	female			male			female			male		
	est	se	pval	est	se	pval	est	se	pval	est	se	pval
	outcome: gross wage category after studying											
mean differences	-0.19	0.33	0.57	0.23	0.27	0.38	0.26	0.28	0.37	-0.09	0.27	0.74
OLS with controls	-0.26	0.33	0.43	0.27	0.29	0.36	0.22	0.28	0.42	-0.18	0.28	0.53
double lasso	-0.36	0.33	0.28	0.23	0.30	0.44	0.18	0.28	0.53	-0.14	0.28	0.63
mean among controls	5.97			7.11			5.97			7.11		
	outcome: gross wage category 3 yrs after studying											
mean differences	0.14	0.33	0.68	0.62	0.32	0.05	0.14	0.32	0.66	-0.21	0.31	0.51
OLS with controls	-0.01	0.35	0.97	0.61	0.34	0.08	0.05	0.32	0.86	-0.18	0.32	0.58
double lasso	-0.07	0.34	0.84	0.60	0.34	0.07	0.05	0.31	0.88	-0.19	0.32	0.54
mean among controls	8.47			9.91			8.47			9.91		

Note: ‘est’, ‘se’, and ‘pval’ reports the ATE estimates, heteroscedasticity robust standard errors, and p-values for the mean difference estimator (‘mean differences’), OLS controlling for *W* (‘OLS with controls’), and doubly robust estimation based on separate lasso estimations of the propensity score and the conditional mean outcome (‘double lasso’). The mean value of the outcome in the control group (‘mean among controls’) is also reported. Dependent variable in survey monthly-wage categories.

Concerning the information treatment, it is worth noting that the gender wage gap of the displayed age categories 20-29 and 30-39 is roughly in line with the average expectational wage gap in the group with the control version of the experiment (see ‘mean among controls’ for males vs. females). However, the expected average wage levels in the control groups are considerably lower than in the graph of the information treatment. The effect estimates (‘est’) suggest that the information treatment increased males’ wage expectations three years after studying by roughly 0.6 categories (or 300 CHF). This finding advances the literature by showing not only that both men and women overestimate their earnings (see [6, 10, 11]); but also we show evidence that male confidence remains even when information is provided, as well as the implications of gender differences in wage expectations for widening the gender expectational gap.

These estimates are marginally statistically significant at the 10% level across the three methods considered, see the heteroscedasticity robust standard errors (‘se’) and p-values (‘pval’). Therefore, the information treatment might increase the expectational gender wage gap, even though effect differences across gender are not statistically significant at the 10% level when running the ‘mean differences’ and ‘OLS with controls’ analyses in a pooled sample of females and males that also includes a treatment-gender interaction as regressor. Furthermore, the information treatment exacerbates over-confidence among males, as early career wages among university graduates are actually lower than expected by both males and females (even without information treatment), see Table 2. In contrast, the treatment did neither statistically significantly affect males’ wage expectations directly after studying (albeit point estimates are again positive) nor female expectations in either period. Secondly, reversing the order of the professional and personal questions did not show any significant impact on wage expectations.

Finally, we address our second question, namely whether or not the inclusion of a broad set of controls, focusing not only on professional preferences but also on personal ones, suffices to account for the direct, unexplained effect of gender on wage expectations commonly found in the literature.

Table 5 reports descriptive statistics for control variables *W*, mediators *X*, and outcomes *Y*, separately by gender *G*. The first 4 columns of Table 5 provide the means of (non-missing values of) the respective variables by gender, mean differences across gender, and the p-values of differences-in-means tests for the original sample. We observe that females and males differ importantly in a range of characteristics like the choice of study program, age, targeted industry and occupation, as well as preferences over job attributes.

**Table 5:** Descriptives and balance tests for covariates and mediators.

	Original sample				After re-weighting	
	mean females	mean males	difference	p-value	difference	p-value
Control variables *W*						
age	22.84	23.44	0.60	0.00	-0.01	0.92
Swiss	0.88	0.88	-0.00	0.92	-0.01	0.62
has siblings	0.92	0.92	-0.00	0.85	-0.03	0.14
mom has higher education	0.23	0.20	-0.03	0.31	0.00	0.93
dad has higher education	0.42	0.37	-0.05	0.13	-0.00	0.98
mum worked full time when I was 4-6	0.21	0.11	-0.09	0.00	0.01	0.57
mum worked part time when I was 4-6	0.44	0.43	-0.01	0.71	-0.00	0.93
wellbeing	2.35	2.36	0.01	0.80	0.04	0.39
home owner	0.44	0.43	-0.01	0.83	-0.04	0.11
treatment: information	0.31	0.33	0.01	0.67	0.02	0.41
treatment: order	0.37	0.31	-0.06	0.07	-0.00	0.91
Mediators *X* (first part): study program, professional plans, intended industry and occupation						
program: business admin	0.71	0.72	0.02	0.58	-0.01	0.70
program: economics	0.03	0.03	0.00	0.96	0.00	0.97
program: communication	0.13	0.03	-0.11	0.00	0.02	0.40
program: business IT	0.07	0.19	0.13	0.00	-0.00	0.93
future plans: work full time	0.64	0.61	-0.04	0.25	0.05	0.09
future plans: education	0.44	0.44	-0.00	0.96	-0.03	0.39
industry: construction	0.01	0.03	0.01	0.10	-0.03	0.09
industry: trade and sales	0.40	0.50	0.11	0.40	0.07	0.22
industry: transport and warehousing	0.02	0.03	0.01	0.23	-0.01	0.66
industry: hospitality and restaurants	0.05	0.01	-0.04	0.00	-0.01	0.31
industry: information and communication	0.38	0.31	-0.08	0.02	0.03	0.36
industry: finance and insurance	0.28	0.44	0.16	0.00	0.03	0.35
industry: consulting	0.12	0.16	0.03	0.14	-0.02	0.44
industry: education and science	0.12	0.08	-0.04	0.05	-0.01	0.62
industry:health and social care	0.06	0.04	-0.02	0.17	-0.01	0.71
occupation: general/strategic management	0.25	0.37	0.12	0.00	-0.01	0.71
occupation: marketing	0.35	0.27	-0.08	0.01	-0.04	0.23
occupation: controlling	0.10	0.15	0.05	0.03	0.00	0.89
occupation: finance	0.20	0.29	0.10	0.00	0.01	0.59
occupation: sales	0.07	0.11	0.04	0.03	-0.02	0.31
occupation: technical/engineering	0.05	0.11	0.06	0.00	-0.04	0.14
occupation: human resources	0.22	0.08	-0.14	0.00	0.00	1.00
position: manager	0.29	0.45	0.17	0.00	0.02	0.50
Mediators *X* (second part): job expectations/views, preferences and plans concerning work and family life						
expect: well paid	3.91	3.90	-0.01	0.84	-0.04	0.43
expect: invest in employees	4.37	4.16	-0.20	0.00	-0.01	0.89
expect: good relations with boss	4.54	4.25	-0.29	0.00	0.10	0.08
expect: job security	4.05	3.73	-0.32	0.00	0.04	0.51
expect: family friendly	3.60	2.81	-0.78	0.00	0.03	0.69
expect: interesting tasks	3.98	3.80	-0.18	0.01	0.04	0.50
expect: identification with work	3.78	3.54	-0.24	0.00	0.06	0.32
expect: priorities are flexible	3.62	3.50	-0.12	0.06	-0.05	0.43
views: fast decision making	2.94	3.04	0.10	0.08	0.01	0.80
views: competitive atmosphere	2.87	3.20	0.33	0.00	-0.06	0.31
views: self responsibility	3.56	3.77	0.21	0.01	0.02	0.81
views:hierarchical structure	2.70	2.94	0.24	0.00	-0.06	0.43
stable partnership in 5-10 years	0.79	0.71	-0.08	0.01	0.02	0.58
preference for family	0.22	0.32	0.10	0.00	0.01	0.64
preference for career	0.02	0.06	0.03	0.01	0.00	0.92
wants children (0 = no, 1 = maybe, 2 = yes)	1.67	1.70	0.03	0.49	-0.04	0.47
Outcomes *Y*						
gross wage category after studying	6.01	7.16	1.15	0.00		
gross wage category 3 yrs after studying	8.57	10.04	1.48	0.00		

Note: Trimming is 0.02 for the balancing tests after re-weighting, such that observations with propensity scores smaller than 0.02 or larger than 0.98 are dropped. Monthly wages expressed in survey categories.

The last 2 columns of Table 5 provide mean differences across gender and p-values after reweighing treated observations by the inverse of the probit estimate of the propensity score Pr(*G* = 1|*X*, *W*) and non-treated observations by the inverse of the estimate of 1 − Pr(*G* = 1|*X*, *W*). (65 observations are dropped due to missing values in *W*, *X*, or *G*). Such reweighing allows assessing whether the propensity score utilized in our IPW procedure (see Table 7 below) successfully balances differences in *W* and *X* across gender as required for evaluating the explained and unexplained components. This indeed appears to be the case when dropping observations with extreme propensity scores below 0.02 or above 0.98 (such that trimming is equal to 0.02 as in Table 7), as most p-values are beyond statistical levels of significance. Only 3 differences in elements of *W* or *X* are statistically significant at the 10% level, while no difference is significant at the 5% level. S1 Fig in S1 Appendix displays the propensity score distributions separately for females and males and demonstrates that they decently overlap.

Table 6 provides the results of the Oaxaca-Blinder decomposition when considering two sets of mediators. In the first approach (‘Mediators *X* (first part)’), we only include those *X* variables that are related to characteristics typically observed and considered in wage decompositions, namely the study program, job or educational plans after finishing the BA studies, as well as the intended industry, occupation, and job position. In our second approach, we add variables that are typically not observed in data sets used for wage decompositions but which are available in our questionnaire (‘Mediators *X* (first and second part)’): preferences about job attributes and the work environment, as well as preferences and plans concerning work and family life. We report the indirect and direct effects (or explained and unexplained components) when considering either females (‘indir.f’, ‘dir.f’), or—as is common in wage decompositions—males (‘indir.m’, ‘dir.m’) as the reference group (*G* = 1). In either case, the direct and indirect effects sum up to the average total gap in wage expectations, defined as the mean difference between males and females (‘total m-f’). The results are presented for two outcomes, namely the expected gross wage category after finishing the studies and three years later. Besides the point estimates (‘est’), standard errors (‘se’) and p-values (‘pval’) based on 499 bootstrap replications are reported.

**Table 6:** Oaxaca-Blinder decomposition.

	Mediators *X* (first part)					Mediators *X* (first and second part)				
	total m-f	indir.f	dir.f	indir.m	dir.m	total m-f	indir.f	dir.f	indir.m	dir.m
	outcome: gross wage category after studying									
est	1.12	0.43	0.69	0.58	0.55	1.07	0.49	0.58	0.69	0.38
se	0.16	0.15	0.22	0.16	0.22	0.16	0.19	0.23	0.19	0.23
pval	0.00	0.00	0.00	0.00	0.01	0.00	0.01	0.01	0.00	0.10
missings		45					72			
	outcome: gross wage category 3 yrs after studying									
est	1.44	0.55	0.89	0.68	0.76	1.41	0.50	0.91	0.78	0.63
se	0.18	0.16	0.23	0.16	0.22	0.19	0.22	0.27	0.21	0.26
pval	0.00	0.00	0.00	0.00	0.00	0.00	0.02	0.00	0.00	0.02
missings		45					72			

Note: ‘total m-f’provides the total expectational wage gap between males and females., ‘indir.f’ and ‘dir.f’ give the indirect (or explained) and direct (or unexplained) components when females are the reference group. ‘indir.m’ and ‘dir.m’ give the respective components when males are the reference group. Point estimates (‘est’) as well as standard errors (‘se’) and p-values (‘pval’) based on 499 bootstrap replications are reported. ‘missings’ provides the numbers of dropped observations due to missingness in variables. Please refer to Table 5 for the list of mediators considered. Dependent variable in survey monthly-wage categories.

The expectational wage gap between males and females amounts to more than one category (or 500 CHF) for wages after studying, and to more than 1.4 categories three years later. This corresponds to roughly 19 and 17% of female average expected wages upon graduation and three years thereafter. The wage gap is driven by both observed characteristics *X* and unexplained factors. Furthermore, when the set of variables in *X* is extended from the subset to all mediators, the magnitude of the indirect effect (or explained component) increases and that of the direct effect (unexplained component) decreases. However, for either outcome, reference group and definition of mediators, both direct and indirect effects remain statistically significant at the 10% level or less, suggesting that even our atypically rich set of mediators cannot fully explain the expectational gender wage gap when resorting to the Oaxaca-Blinder decomposition. ([23] also decompose the expectational gender wage gap trough an Oaxaca-Blinder decomposition and present their results with the female as the reference. There, explained and unexplained components of the gap are roughly equal in size. In our decomposition, the unexplained part is larger (e.g. ‘dir.f’ is about 20% larger than ‘indir.f’ for the graduation time horizon—0.58 compared to 0.49—and about 80% as large three years afterwards, when all mediators are included—0.91 compared to 0.5)).

Table 7 reports the results for IPW, which allows controlling for potential confounders *W* and relaxing linearity assumptions. We to this end use the ‘medweight’ command of the ‘causalweight’ package by [61] for the statistical software ‘R’, with trimming set to 0.02 and 499 bootstrap replications for the estimation of the standard errors. Even though the precision of estimation is somewhat lower than before, the results bear qualitative similarities to the Oaxaca-Blinder decomposition in that both direct and indirect effects generally remain important for explaining the expectational wage gap. However, focusing on the more commonly used wage decomposition in the literature (which takes the male wage as the reference), the unexplained part of the gender gap in wage expectations (‘dir.m’) is greatly reduced when all mediators are included and loses statistical significance (p-value of 0.39 for wage expectations upon graduation and 0.09 for three years on). Conversely, the magnitude of the indirect effect increases (when we go from a subset of mediators to the full sample) and retains statistical significance. Using a larger trimming threshold of 0.04 results in quite stable results (S1 Table in S1 Appendix).

**Table 7:** Decomposition with trimming equal to 0.02.

	Mediators *X* (first part)					Mediators *X* (first and second part)				
	total m-f	indir.f	dir.f	indir.m	dir.m	total m-f	indir.f	dir.f	indir.m	dir.m
	outcome: gross wage category after studying									
est	1.06	0.37	0.69	0.56	0.50	0.94	0.45	0.49	0.65	0.29
se	0.17	0.18	0.24	0.17	0.22	0.18	0.24	0.27	0.30	0.34
pval	0.00	0.04	0.01	0.00	0.02	0.00	0.06	0.07	0.03	0.39
missings / trimmed		45	/	3			72	/	25	
	outcome: gross wage category 3 yrs after studying									
est	1.37	0.50	0.87	0.67	0.71	1.29	0.48	0.81	0.81	0.48
se	0.17	0.19	0.25	0.16	0.21	0.19	0.31	0.34	0.25	0.28
pval	0.00	0.01	0.00	0.00	0.00	0.00	0.12	0.02	0.00	0.09
missings / trimmed		45	/	4			72	/	20	

Note: ‘total m-f’provides the total expectational wage gap between males and females., ‘indir.f’ and ‘dir.f’ give the indirect (or explained) and direct (or unexplained) components when females are the reference group. ‘indir.m’ and ‘dir.m’ give the respective components when males are the reference group. Point estimates (‘est’) as well as standard errors (‘se’) and p-values (‘pval’) based on 499 bootstrap replications are reported. ‘missings’ and ‘trimmed’ provide the numbers of dropped observations due to missingness in variables and extreme propensity scores, respectively. Refer to Table 5 for the list of mediators considered. Dependent variable in survey monthly-wage categories.

We additionally performed the Oaxaca-Blinder decomposition as well as the IPW approach (with a trimming threshold of 0.02 and 0.04) when considering the logarithm of the expected wage category as the outcome (S2–S4 Tables in S1 Appendix). The results of the Oaxaca-Blinder decomposition are mostly qualitatively unaffected by this transformation of the outcome. As for the IPW decomposition, using the log expected wage as an outcome reinforces the relevance of considering the broader set of mediators in our dataset as the direct unexplained effect of gender becomes very small and statistically insignificant when males are the reference both for expectations at graduation and three years thereafter and for either trimming setting. The bulk of the evidence thus points toward a mitigated direct effect of gender on wage expectations once professional and personal preferences are taken into consideration.

We take an additional step in our decomposition of gender differences in wage expectations by considering yet another partition of the set of mediators. Specifically, we consider now the case when only professional mediators are included in the analysis, and then add those that are exclusively of a personal nature. The latter are listed in the bottom four rows of Table 5, middle section (Mediators *X* (second part)), from ‘stable partnership’ to ‘wants children.’ The professional ones are all of the remaining in the rows above. This new partition allows us to assess the quantitative and qualitative relevance of professional versus personal factors in the determination of gender differences in wage expectations.

Table 8 shows the results for wage expectations upon graduation (and trimming set to 0.02). Top and bottom panels on the left show results including only professional mediators whereas the panels on the right include all mediators and simply replicate the results of the corresponding panels (top and bottom right) in Table 7 for visual convenience. Table 9 further presents the results in Table 8 but now in percentage form: each number in Table 9 is presented as a fraction of the full effect (‘total m-f’) in the corresponding panel from Table 8.

**Table 8:** Disentangling the contributions of professional and personal mediators (trimming equal to 0.02).

	Professional Mediators Only					All Mediators				
	total m-f	indir.f	dir.f	indir.m	dir.m	total m-f	indir.f	dir.f	indir.m	dir.m
	outcome: gross wage category after studying									
est	1.01	0.38	0.63	0.54	0.47	0.94	0.45	0.49	0.65	0.29
se	0.17	0.20	0.25	0.18	0.23	0.18	0.24	0.27	0.30	0.34
pval	0.00	0.06	0.01	0.00	0.04	0.00	0.06	0.07	0.03	0.39
missings / trimmed		72	/	8			72	/	25	
	outcome: gross wage category 3 yrs after studying									
est	1.31	0.34	0.97	0.65	0.67	1.29	0.48	0.81	0.81	0.48
se	0.19	0.26	0.31	0.21	0.25	0.19	0.31	0.34	0.25	0.28
pval	0.00	0.19	0.00	0.00	0.01	0.00	0.12	0.02	0.00	0.09
missings / trimmed		45	/	4			72	/	20	

Note: ‘total m-f’provides the total expectational wage gap between males and females., ‘indir.f’ and ‘dir.f’ give the indirect (or explained) and direct (or unexplained) components when females are the reference group. ‘indir.m’ and ‘dir.m’ give the respective components when males are the reference group. Point estimates (‘est’) as well as standard errors (‘se’) and p-values (‘pval’) based on 499 bootstrap replications are reported. ‘missings’ and ‘trimmed’ provide the numbers of dropped observations due to missingness in variables and extreme propensity scores, respectively. Personal mediators: those listed in the bottom four rows of Table 5; professional mediators are all of those remaining in the same table. Dependent variable is survey monthly-wage categories.

**Table 9:** Disentangling the contributions of professional and personal mediators in percent of the total effect (trimming equal to 0.02).

	Professional Mediators Only (in% of total m-f)					All Mediators (in% of total m-f)				
	total m-f	indir.f	dir.f	indir.m	dir.m	total m-f	indir.f	dir.f	indir.m	dir.m
	outcome: gross wage category after studying									
est	100	37.62	62.38	53.47	46.53	100	47.87	52.13	69.15	30.85
se	0.17	0.20	0.25	0.18	0.23	0.18	0.24	0.27	0.30	0.34
pval	0.00	0.06	0.01	0.00	0.04	0.00	0.06	0.07	0.03	0.39
missings / trimmed		72	/	8			72	/	25	
	outcome: gross wage category 3 yrs after studying									
est	100	25.95	74.05	49.62	51.15	100	37.21	62.79	62.79	37.21
se	0.19	0.26	0.31	0.21	0.25	0.19	0.31	0.34	0.25	0.28
pval	0.00	0.19	0.00	0.00	0.01	0.00	0.12	0.02	0.00	0.09
missings / trimmed		45	/	4			72	/	20	

Note: This table replicates Table 8 while recalculating the individual effects in percentage of the corresponding average total effect indicated as ‘total m-f’. Please refer to the notes of Table 8 for additional information.

Let us now re-examine the impact of gender on wage expectations at graduation. When considering only professional mediators (Table 8), all direct and indirect effects are statistically significant. Having the male wage as the reference, direct and indirect effects are of similar magnitude (0.47 and 0.54 respectively). When personal mediators are added to the analysis, the indirect effect increases and the direct effect declines; further, the direct effect when the male wage is the reference loses significance. As stated, Table 9 recasts the contribution of direct and indirect effects as percentages of the total effect. Comparing the left and right panels of this table, we see that the inclusion of personal mediators raises the share of the indirect effect (male wage is the reference) from 53.5% to 69.2% of the total effect, while the share accounted for by the direct effect declines from 46.5% to 30.9%. Thus, personal mediators contribute to a sizeable quantitative reduction and to a loss in statistical significance in the unexplained part of the gender expectational wage gap. (Personal mediators alone explain virtually nothing of the indirect effect of gender on wage expectations (not shown). When the decomposition taking the male as the reference is considered, the coefficient on the indirect effect is -.04 (p-value of 0.49) and the coefficient on the direct effect is 1.4 (p-value of 0.00), for a total effect of 1.35). As the bottom panels in Tables 8 and 9 show, results for the longer time horizon are qualitatively similar. S5 and S6 Tables in S1 Appendix repeat the results above for trimming set to 0.04 with similar conclusions. (Under the Oaxaca-Blinder decomposition (not shown), there is virtually no numerical change associated with the inclusion of personal effects for wage expectations upon graduation. There is a small change in the longer time horizon augmenting the indirect effect and reducing the direct one, when the male wage is the reference).

## 6 Conclusion

Using novel survey data from students from the Business School of the Bern University of Applied Science (BUAS) and the Faculty of Economic and Social Sciences of the University of Fribourg, this paper advances the literature related to gender differences in wage expectations in two specific ways. First, it determines whether these gender differences are rational by comparing expected wages from our respondents to realized wages from comparable graduates; and, further, by investigating how respondents adjust their wage expectations when information about actual wages is provided. Second, using an inverse probability weighting method in the context of causal mediation, it examines whether the consideration of a rich set of professional and personal controls accounts for the difference in wage expectations across gender.

In line with the literature, we confirm the presence of gender differences in wage expectations in our survey results. The difference between male and female expected wages is about one salary class (CHF500) upon graduation and roughly 1.4 salary classes three years thereafter (roughly 19 and 17% of female average expected wages, respectively). The evidence suggests that both males and females overestimate their wages relative to realized wages from comparable graduates. Further, results from an information intervention—about median wages earned in Switzerland—show that males alone (incorrectly) revise their expected wages upward by about 0.6 of a salary class (CHF300) when forecasting wages three years after graduation. This is possibly the result of over-confidence.

Using mediation analysis (which permits explicating endogeneity issues), we find that the inclusion of a rich set of personal and professional mediators—not commonly included in survey data—greatly reduces the direct, unexplained effect of gender on wage expectations. While personal mediators alone do not contribute to the indirect effect of gender on wage expectations, when added to professional mediators they lead to a reduction of about 30% in the contribution of the direct effect of gender on wage expectations and to a similar increase in the indirect effect (when the decomposition of these effects takes the male as the reference). Further, when professional and personal mediators are jointly considered, the direct, unexplained effect of gender is greatly attenuated, both in size as well as in statistical significance. Nonetheless, a non-negligible and statistically significant direct (or unexplained) effect of gender on wage expectations remains in several, but not all specifications. Our results are stable under different specifications and trimming thresholds.

## Supporting information

S1 Appendix (PDF)
