# Supplementary material for: Gender differences in wage expectations
Source: PLoS One. 2021 Jun 2;16(6):e0250892. doi: 10.1371/journal.pone.0250892 (PMC8172067; doi:10.1371/journal.pone.0250892)
Supplement: S1 Appendix — (PDF) [file pone.0250892.s001.pdf]

A Appendix

A.1 Tables and figures

S1 Table. Decomposition with trimming equal to 0.04

	subset of mediators					all mediators				
	total m-f	indir.f	dir.f	indir.m	dir.m	total m-f	indir.f	dir.f	indir.m	dir.m
	outcome: gross wage category after studying									
est	1.05	0.37	0.68	0.55	0.50	0.90	0.35	0.55	0.64	0.26
se	0.17	0.17	0.24	0.16	0.22	0.19	0.23	0.29	0.25	0.29
p-value	0.00	0.03	0.00	0.00	0.03	0.00	0.12	0.06	0.01	0.36
missings / trimmed		45	/	10			72	/	61	
	outcome: gross wage category 3 yrs after studying									
est	1.36	0.49	0.86	0.66	0.70	1.18	0.48	0.71	0.72	0.46
se	0.21	0.18	0.27	0.17	0.25	0.22	0.25	0.33	0.24	0.28
p-value	0.00	0.00	0.00	0.00	0.00	0.00	0.06	0.03	0.00	0.10
missings / trimmed		45	/	8			72	/	64	

Note: 'total m-f'provides the total expectational wage gap between males and females. , 'indir.f' and 'dir.f' give the indirect (or explained) and direct (or unexplained) components when females are the reference group. 'indir.m' and 'dir.m' give the respective components when males are the reference group. Point estimates ('est') as well as standard errors ('se') and p-values ('pval') based on 499 bootstrap replications are reported. 'missings' and 'trimmed' provide the numbers of dropped observations due to missingness in variables and extreme propensity scores, respectively. Dependent variable is survey monthly-wage categories.

S1 Fig. **Common support for estimated** $\Pr(G = 1|W, X)$

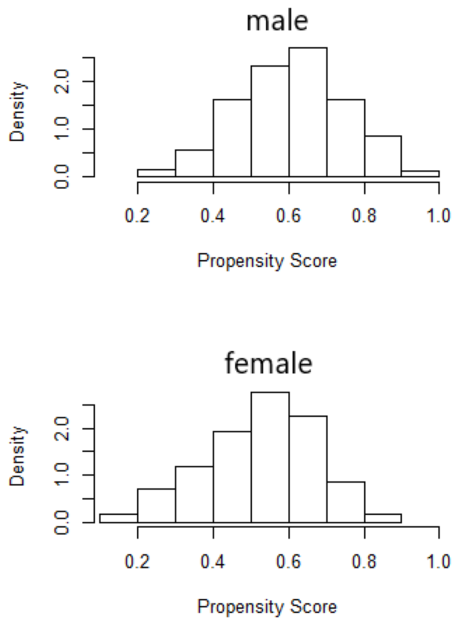

S2 **Table. Oaxaca Blinder decomposition, outcome is log wage**

	subset of mediators					all mediators				
	total m-f	indir.f	dir.f	indir.m	dir.m	total m-f	indir.f	dir.f	indir.m	dir.m
	outcome: gross wage category after studying									
est	0.16	0.07	0.09	0.09	0.07	0.15	0.07	0.07	0.11	0.03
se	0.03	0.02	0.03	0.03	0.04	0.03	0.03	0.04	0.03	0.04
p-value	0.00	0.00	0.01	0.00	0.05	0.00	0.02	0.06	0.00	0.42
missings		45					72			
	outcome: gross wage category 3 yrs after studying									
est	0.13	0.06	0.07	0.06	0.07	0.13	0.05	0.08	0.08	0.05
se	0.02	0.02	0.02	0.02	0.03	0.02	0.02	0.03	0.02	0.03
p-value	0.00	0.00	0.00	0.00	0.01	0.00	0.02	0.01	0.00	0.07
missings		45					72			

Note: ‘total m-f’provides the total expectational wage gap between males and females. , ‘indir.f’ and ‘dir.f’ give the indirect (or explained) and direct (or unexplained) components when females are the reference group. ‘indir.m’ and ‘dir.m’ give the respective components when males are the reference group. Point estimates (‘est’) as well as standard errors (‘se’) and p-values (‘pval’) based on 499 bootstrap replications are reported. ‘missings’ provides the numbers of dropped observations due to missingness in variables. Dependent variable is survey monthly-wage categories.

S3 Table. Decomposition with trimming equal to 0.02, outcome is log wage

	subset of mediators					all mediators				
	total m-f	indir.f	dir.f	indir.m	dir.m	total m-f	indir.f	dir.f	indir.m	dir.m
	outcome: gross wage category after studying									
est	0.14	0.05	0.09	0.08	0.06	0.12	0.06	0.06	0.14	-0.01
se	0.03	0.02	0.03	0.03	0.04	0.03	0.03	0.04	0.07	0.07
p-value	0.00	0.04	0.01	0.01	0.15	0.00	0.05	0.12	0.05	0.87
missings / trimmed		45	/	3			72	/	25	
	outcome: gross wage category 3 yrs after studying									
est	0.13	0.05	0.08	0.06	0.06	0.12	0.05	0.07	0.08	0.04
se	0.02	0.02	0.03	0.02	0.03	0.02	0.03	0.03	0.03	0.03
p-value	0.00	0.01	0.00	0.00	0.02	0.00	0.11	0.04	0.00	0.16
missings / trimmed		45	/	4			72	/	20	

Note: 'total m-f'provides the total expectational wage gap between males and females. , 'indir.f' and 'dir.f' give the indirect (or explained) and direct (or unexplained) components when females are the reference group. 'indir.m' and 'dir.m' give the respective components when males are the reference group. Point estimates ('est') as well as standard errors ('se') and p-values ('pval') based on 499 bootstrap replications are reported. 'missings' and 'trimmed' provide the numbers of dropped observations due to missingness in variables and extreme propensity scores, respectively. Dependent variable is survey monthly-wage categories.

S4 Table. Decomposition with trimming equal to 0.04, outcome is log wage

	subset of mediators					all mediators				
	total m-f	indir.f	dir.f	indir.m	dir.m	total m-f	indir.f	dir.f	indir.m	dir.m
	outcome: gross wage category after studying									
est	0.14	0.05	0.09	0.08	0.06	0.12	0.04	0.07	0.13	-0.02
se	0.02	0.02	0.03	0.03	0.04	0.03	0.03	0.04	0.05	0.06
p-value	0.00	0.03	0.01	0.00	0.11	0.00	0.14	0.05	0.01	0.76
missings / trimmed		45	/	10			72	/	61	
	outcome: gross wage category 3 yrs after studying									
est	0.12	0.05	0.07	0.06	0.06	0.11	0.04	0.06	0.07	0.04
se	0.02	0.02	0.02	0.02	0.03	0.02	0.03	0.03	0.02	0.03
p-value	0.00	0.01	0.00	0.00	0.01	0.00	0.10	0.05	0.01	0.18
missings / trimmed		45	/	8			72	/	64	

Note: 'total m-f'provides the total expectational wage gap between males and females. , 'indir.f' and 'dir.f' give the indirect (or explained) and direct (or unexplained) components when females are the reference group. 'indir.m' and 'dir.m' give the respective components when males are the reference group. Point estimates ('est') as well as standard errors ('se') and p-values ('pval') based on 499 bootstrap replications are reported. 'missings' and 'trimmed' provide the numbers of dropped observations due to missingness in variables and extreme propensity scores, respectively. Dependent variable is survey monthly-wage categories.

S5 Table. Disentangling the Contributions of Professional and Personal Mediators (trimming equal to 0.04)

	Professional Mediators Only					All Mediators				
	total m-f	indir.f	dir.f	indir.m	dir.m	total m-f	indir.f	dir.f	indir.m	dir.m
	outcome: gross wage category after studying									
est	0.95	0.38	0.56	0.48	0.47	0.90	0.35	0.55	0.64	0.26
se	0.18	0.19	0.24	0.18	0.22	0.18	0.24	0.30	0.26	0.31
pval	0.00	0.04	0.02	0.01	0.04	0.00	0.14	0.06	0.01	0.39
missings / trimmed		72	/	31			72	/	61	
	outcome: gross wage category 3 yrs after studying									
est	1.23	0.33	0.90	0.57	0.65	1.18	0.48	0.71	0.72	0.46
se	0.20	0.25	0.30	0.21	0.25	0.20	0.26	0.31	0.24	0.27
pval	0.00	0.18	0.00	0.01	0.01	0.00	0.06	0.02	0.00	0.09
missings / trimmed		72	/	28			72	/	64	

Note: 'total m-f'provides the total expectational wage gap between males and females. , 'indir.f' and 'dir.f' give the indirect (or explained) and direct (or unexplained) components when females are the reference group. 'indir.m' and 'dir.m' give the respective components when males are the reference group. Point estimates ('est') as well as standard errors ('se') and p-values ('pval') based on 499 bootstrap replications are reported. 'missings' and 'trimmed' provide the numbers of dropped observations due to missingness in variables and extreme propensity scores, respectively. Personal mediators: those listed in the bottom four rows of Table 5; professional mediators are all of those remaining in the same table. Dependent variable is survey monthly-wage categories.

S6 Table. Disentangling the Contributions of Professional and Personal Mediators in Percent of the Average Total Effect (trimming equal to 0.04)

	**Professional Mediators Only (in % of total m-f)**					**All Mediators (in % of total m-f)**				
	total m-f	indir.f	dir.f	indir.m	dir.m	total m-f	indir.f	dir.f	indir.m	dir.m
	outcome: gross wage category after studying									
est	100	40.00	58.95	50.53	49.47	100	38.89	61.11	71.11	28.89
se	0.18	0.19	0.24	0.18	0.22	0.18	0.24	0.30	0.26	0.31
pval	0.00	0.04	0.02	0.01	0.04	0.00	0.14	0.06	0.01	0.39
missings / trimmed		72	/	31			72	/	61	
	outcome: gross wage category 3 yrs after studying									
est	100	26.83	73.17	46.34	52.85	100	40.68	60.17	61.02	38.98
se	0.20	0.25	0.30	0.21	0.25	0.20	0.26	0.31	0.24	0.27
pval	0.00	0.18	0.00	0.01	0.01	0.00	0.06	0.02	0.00	0.09
missings / trimmed		72	/	28			72	/	64	

Note: Note: This table replicates S5 Table while recalculating the individual effects in percentage of the corresponding average total effect indicated as 'total m-f'. Please refer to the notes of S5 Table for additional information.

A.2 Questionnaire

698

Dear students

The answers to the following questions are to be used for research. There is no identifier that could trace the questions to any particular individual, so your answers will remain strictly confidential.

The survey is fully voluntary. If you choose to answer it, know that I do appreciate your taking the time.

Thank you and best regards,
Ana Fernandes

v_p

1

**A1**	**General questions about yourself**
A1.1	How old are you? ______
A1.2	Your gender is: □ Female □ Male
A1.3	What is your nationality (indicate the country you feel closer to, if multiple are possible)? ____
A1.4	Which degree programme are you taking at the Bern University of Applied Sciences? □ Bachelor of ____________ I □ Master of ____________ I □ ____________
A1.5	Which study model have you chosen? □ Full-time degree programme I □ Part-time degree programme I □ ____________
**A2**	**Questions about your professional path**
	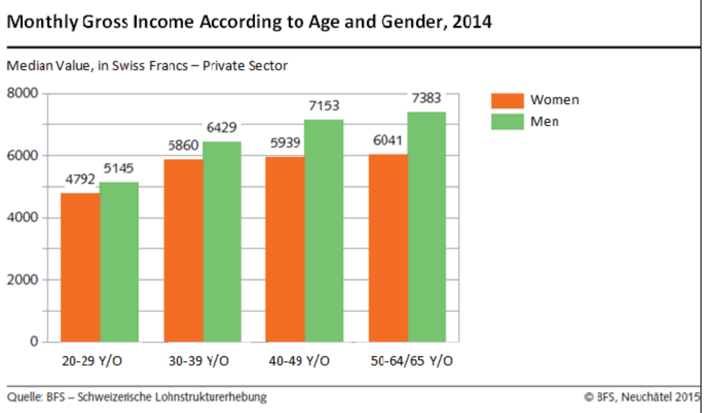
A2.1	Which statement best describes you at the moment?<br>□ You already have work experience (full-time or part-time)<br>□ You have held odd jobs to supplement income or not worked at all<br>□ Other ______________________________
A2.2	What are your plans regarding work or study once you complete the degree you are currently enrolled in? **Multiple answers possible**:<br>□ Work full-time □ Work part-time □ Further education (MA, or another advanced degree, or...)<br>□ Do not intend to seek employment □ Other ______________________________

2

A2.3	Consider the following company and job attributes. Please assign a grade from 1 to 5 to each one, with 5 being highly valued and 1 being not important.<br><br>__ Company pays well<br><br>__ Company invests in people and in their careers<br><br>__ Flexible working conditions with respect to schedule and location<br><br>__ Pleasant relations with boss and colleagues<br><br>__ Job security<br><br>__ Supportive policies to accommodate maternity/paternity<br><br>__ Daily tasks are intellectually stimulating<br><br>__ Your work in the company contributes to a cause that is important to you<br><br>__ Flexibility in setting priorities and tasks<br><br>__ The commuting time between your home and the office is short
A2.4	Consider the following company and job attributes. Please assign a grade from 1 to 5 to each one, with 5 being very good and 1 being very bad.<br><br>__ Long hours<br><br>__ Repetitive<br><br>__ Need to systematically make decisions in a very short time<br><br>__ Mistakes would impact negatively the well-being of many people<br><br>__ The environment is very competitive<br><br>__ Personal accountability for successes and mistakes<br><br>__ Hierarchical firm structure
A2.5	What are your expected monthly gross earnings (no taxes deducted) when you graduate from the current studies (in Swiss Francs)?<br><br>□ Below 3'500 □ 3'500-4'000 □ 4'000-4'500 □ 4'500-5'000 □ 5'000-5'500 □ 5'500-6'000<br><br>□ 6'000-6'500 □ 6'500-7'000 □ 7'000-7'500 □ 7'500-8'000 □ 8'000-8'500 □ 8'500-9'000<br><br>□ 9'000- 9'500 □ 9'500-10'000 □ 10'000-10'500 □ 10'500-11'000 □ More than 11'000
A2.6	What are your expected monthly gross earnings (no taxes deducted) 3 years after graduation (in Swiss Francs)?<br><br>□ Below 3'500 □ 3'500-4'000 □ 4'000-4'500 □ 4'500-5'000 □ 5'000-5'500 □ 5'500-6'000<br><br>□ 6'000-6'500 □ 6'500-7'000 □ 7'000-7'500 □ 7'500-8'000 □ 8'000-8'500 □ 8'500-9'000<br><br>□ 9'000- 9'500 □ 9'500-10'000 □ 10'000-10'500 □ 10'500-11'000 □ More than 11'000

3

A2.7	Set your salary at graduation at 100. How much do you think will be earned by a student with the same characteristics as yours but of the other gender?<br>Salary at Graduation<br>□ 50 □ 60 □ 70 □ 80 □ 90 □ 100 □ 110 □ 120 □ 130 □ 140 □ 150<br>If possible, please say why____
A2.8	Set your salary *10 years after* graduation at 100. How much do you think will be earned by a student with the same characteristics as yours but of the other gender?<br>Salary 10 Y After Graduation<br>□ 50 □ 60 □ 70 □ 80 □ 90 □ 100 □ 110 □ 120 □ 130 □ 140 □ 150<br>If possible, please say why ____
A2.9	In which industry do you expect to be working upon completion of your degree?<br>□ Agriculture, forestry, fishing, mining □ Manufacturing □ Construction □ Trade and Sales<br>□ Transportation and storage □ Accommodation and food service □ Information and communication<br>□ Finance and insurance □ Real estate □ Consulting □ Public Administration<br>□ Education and Science □ Health care and social care □ Arts, entertainment and recreation<br>□ Other _______________________________________ □ Don't know
A2.10	In which occupation do you expect to be working upon completion of your degree?<br>□ General and strategic management □ Marketing □ Controlling □ Finance<br>□ Research and Development □ Sales □ Logistics □ Production<br>□ Technical support and engineering □ HR □ Unskilled occupation<br>□ Other ____ □ Don't know
A2.11	In your future professional career, which role would you prefer to have:<br>□ A management/leadership position in a company (for example CEO)?<br>□ A specialist/supporting position (for example the head of a cabinet providing specialized support to the CEO)?<br>□ Not sure yet<br>□ If you chose one of the first two options above, please explain why you prefer one over the other<br>____
A3	**Questions about family and other personal dimensions**
A3.1	In the next 5 to 10 years, do you see yourself in a stable partner relationship?<br>□ Yes □ No □ Not sure

A3.2	Would you like to have children eventually? If your answer is "yes," please indicate how many.<br>□ No □ Yes □ Not sure (If yes) How many? _____
A3.3	If you were in a stable partner relationship with children in the next 5 to 10 years, which employment type would you prefer:<br>□ Work full-time □ Work part-time □ Not work □ Other ___________________________
A3.4	If you were in a stable partner relationship with children in the next 5 to 10 years, which employment type would you prefer your partner to have:<br>□ Work full-time □ Work part-time □ Not work □ The employment status preferred by the partner<br>□ Other____
A3.5	When you were a young child of pre-Kindergarten age (roughly ages 0-4), what was the employment status of your parents in general?<br>□ My mother did not work □ My father did not work<br>□ My mother worked part-time □ My father worked part-time<br>□ My mother worked full-time □ My father worked full-time<br>□ Other: ______________ □ Other: ______________
A3.6	When you were a young child of Kindergarten age (roughly ages from 4-6), what was the employment status of your parents in general?<br>□ My mother did not work □ My father did not work<br>□ My mother worked part-time □ My father worked part-time<br>□ My mother worked full-time □ My father worked full-time<br>□ Other: ______________ □ Other: ______________
A3.7	During your first school years (1st to 6th grade), what was the employment status of your parents in general?<br>□ My mother did not work □ My father did not work<br>□ My mother worked part-time □ My father worked part-time<br>□ My mother worked full-time □ My father worked full-time<br>□ Other: ______________ □ Other: ______________
A3.8	Which sentence describes you best?<br>□ Family life is important to me<br>□ Family life is important to me but so is my professional career<br>□ I value my professional career more than I value my family life

A3.9	Please tell us about your family members by indicating the number of relatives that you have of each type below.<br><br>Siblings ___ Living parents or step parents ___ Living grandparents____<br><br>Partner/girlfriend/boyfriend____ Own children____
A3.10	Please answer this question if you have siblings. How many of your siblings are older than you?<br><br>I have ___ sisters who are older than me. I have ___ brothers who are older than me.
A3.11	How many people do you share your place of residence with? ________<br><br>If you do not live alone, please specify who you are living with (parents, siblings, ...)<br><br>____
A3.12	What type of residence do you live in?<br><br>□ Rented flat □ Rented house □ Own flat □ Own house □ Other _________________
A3.13	What is the highest educational attainment of your parents:<br><br>Mother / Father<br><br>Compulsory schooling (up to lower secondary schooling) or less □ □<br><br>Apprenticeship or vocational degree □ □<br><br>Upper secondary schooling (including high school) □ □<br><br>University (Bachelor degree) □ □<br><br>University (Masters degree) □ □<br><br>University (PhD/doctorate) □ □<br><br>Another (please specify) □ □<br><br>_____________ _____________
A3.14	How would you evaluate your material well-being compared to the average person in Switzerland? I am doing:<br><br>Much worse than average person □ / Worse than average person □ / Roughly the same as average person □ / Better than average person □ / Much better than average person □

6
